# Noninvasive differentiation of upper tract urothelial carcinoma and renal cell carcinoma in challenging renal lesions using urinary DNA methylation

**DOI:** 10.1002/bco2.70265

**Published:** 2026-08-04

**Authors:** Ernest Kaufmann, Kilian Röthlin, Philipp Baumeister, Agostino Mattei, Paolo Piatti, Yap Ching Chew, Christian Daniel Fankhauser

**Affiliations:** ^1^ Faculty of Health Sciences and Medicine University of Lucerne Lucerne Switzerland; ^2^ Clinic for Urology University Teaching and Research Hospital of the University of Lucerne Lucerne Switzerland; ^3^ Zymo Research Corp Irvine California USA; ^4^ Pangea Laboratory LLC Tustin California USA; ^5^ University of Zurich Zurich Switzerland; ^6^ Division of Urology, Department of Surgery Cantonal Hospital of Winterthur Winterthur Switzerland

**Keywords:** DNA methylation, non‐invasive testing, RCC, renal cell carcinoma, upper tract urothelial carcinoma, urine marker, UTUC

Upper tract urothelial carcinoma (UTUC) and renal cell carcinoma (RCC) may present with similar radiographic findings, making the diagnostic evaluation of indeterminate lesions challenging.^1^ The distinction is clinically important because management diverges substantially: UTUC typically requires radical nephroureterectomy with bladder‐cuff excision and intravesical chemotherapy instillation, whereas RCC is generally managed by (partial) nephrectomy.^2^, ^3^ When imaging is inconclusive, patients frequently undergo additional invasive work‐up, such as ureterorenoscopy (URS) with biopsy or percutaneous renal biopsy. URS remains the diagnostic reference standard for suspected UTUC but carries risk of procedure‐related morbidity including ureteric injury, infection, haematuria, and potential intravesical recurrence, and its diagnostic yield is imperfect.^4^, ^5^ Voided urinary cytology is noninvasive but has limited sensitivity (10%–70%), even when selective upper tract sampling is used.^6^ The urine‐based DNA methylation assay Bladder CARE™ has demonstrated high accuracy for UTUC detection,^7^, ^8^ but its ability to distinguish UTUC from RCC in patients with renal or renal‐pelvic lesions has not yet been investigated. We addressed this question in a small, exploratory, post hoc analysis.

Between December 2023 and August 2025, we prospectively collected urine from patients presenting with renal or renal‐pelvic lesions suspicious for malignancy in whom cross‐sectional imaging did not reliably differentiate UTUC from RCC. Patients with radiologically unequivocal exophytic renal masses were not included. For this post hoc analysis we retained only patients with histologically confirmed UTUC or RCC and an available preoperative Bladder CARE™ result. Assay results were available to treating physicians but were not used to guide management; subsequent work‐up and treatment followed clinical judgment, and histopathology of biopsy or surgical specimens served as the reference standard. The Bladder CARE™ assay measures methylation levels of three urothelial‐cancer DNA biomarkers (TRNA‐Cys, SIM2, and NX1‐1) and reports a continuous Bladder CARE™ Index (BCI). A subset of patients overlapped with a previously published UTUC validation cohort^7^; the present analysis addresses a distinct question and adds further patients. Urinary cytology was evaluated using the Paris System for Reporting Urinary Cytology. Samples categorized as ‘Negative for High Grade Urothelial Carcinoma’ (NHGUC) or ‘Atypical Urothelial Cells’ (AUC) were considered cytology‐negative, whereas ‘Suspicious for High Grade Urothelial Carcinoma’ (SHGUC) and ‘High Grade Urothelial Carcinoma’ (HGUC) were classified as positive. The study was approved by the responsible ethics committee (BASEC‐ID 2025‐01778), and all participants gave written informed consent. Given the exploratory design and the rarity of the scenario, no a priori sample‐size calculation was performed and analyses were primarily descriptive. Group differences in BCI were assessed with the Mann–Whitney U test, discrimination with receiver‐operating‐characteristic (ROC) analysis, and an exploratory optimal cut‐off with the Youden index. Statistical analyses were performed using R Studio (R Core Team, 2022, Version 2023.03.0+386).

Eighteen patients with histologically confirmed disease were included (11 UTUC, 7 RCC); median age was 70 years (IQR 58–76). Most UTUC patients underwent radical nephroureterectomy (10/11) and most RCC patients (partial) nephrectomy (5/7). The cohort reflected the real‐world diagnostic dilemma: several RCC cases initially entered an UTUC work‐up because imaging was inconclusive, including three patients who underwent URS (two with benign biopsies later confirmed as RCC on percutaneous sampling) and one patient underwent radical nephroureterectomy for presumed UTUC with final pathology showing pT3 RCC. Conventional urinary cytology was negative in all patients and did not separate the groups. Median BCI values were markedly higher in UTUC than in RCC (19.2 [IQR 8.9–71.5] vs. 1.9 [IQR 0.55–2.75], *p* = 0.002; Figure 1), and ROC analysis yielded an area under the curve of 0.95 (95% CI 0.84–1.00). The exploratory Youden‐derived cut‐off of 4.8 correctly classified 10/11 UTUC and all seven RCC cases, corresponding to a sensitivity of 91% (95% CI 59%–100%), specificity of 100% (95% CI 59%–100%), positive predictive value of 100%, negative predictive value of 88%, and overall accuracy of 94% (17/18). No RCC case produced a false‐positive result. Within the UTUC cases, BCI did not differ significantly between low‐grade and high‐grade tumours (10.5 vs. 28.0; *p* = 0.79).

**FIGURE 1:**
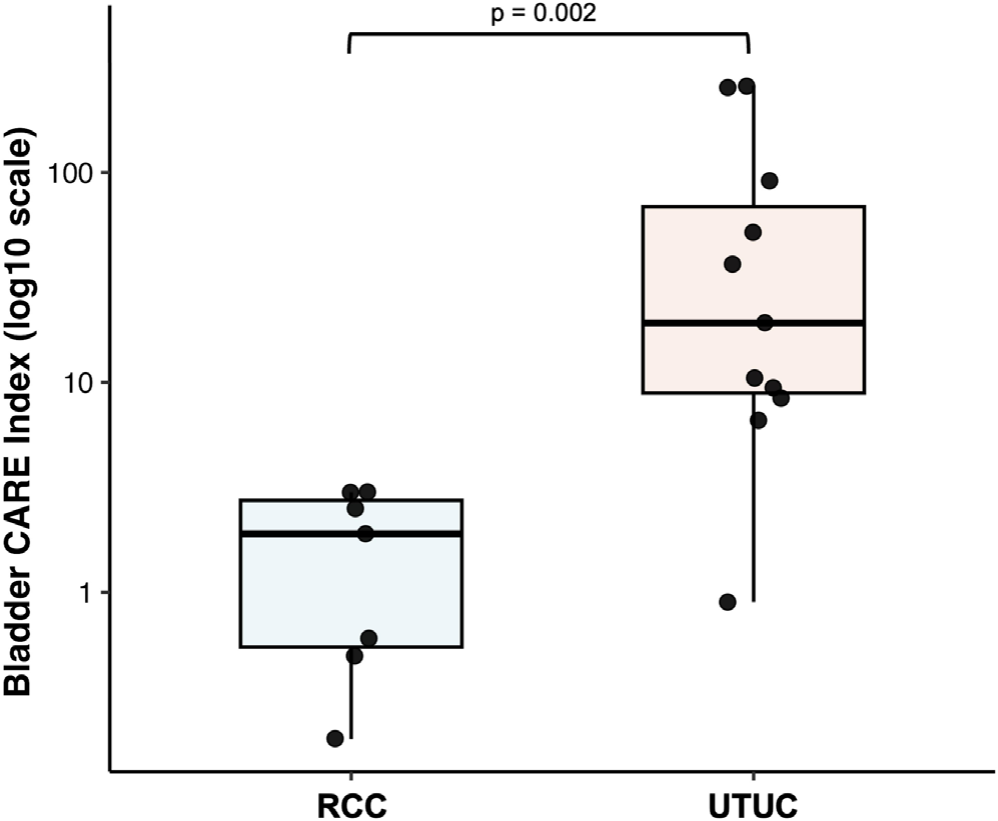
Boxplot of bladder CARE values stratified by histology. Bladder CARE values displayed on a log10 scale and stratified by histology (RCC vs. UTUC). Median BCI values were significantly higher in UTUC than in RCC (19.2 vs. 1.9, *p* = 0.002). Individual data points are overlaid. BCI: Badder CARE Index, RCC: renal cell carcinoma, UTUC: upper tract urothelial carcinoma.

In this exploratory series the Bladder CARE™ assay separated UTUC from RCC with a high AUC and no false‐positive RCC results, in a clinical setting where imaging and cytology were unhelpful. A plausible biological explanation is that the assay targets methylation changes in urothelial‐cancer DNA shed into the urine; RCC, which is of nonurothelial origin, would not be expected to release these markers, consistent with the consistently low BCI values observed in the RCC group. The strongest illustration of the underlying problem comes from the cohort itself: several patients ultimately found to have RCC were initially managed as UTUC, undergoing diagnostic URS and, in one case, nephroureterectomy. Such misdirected pathways expose patients to avoidable procedural risk and delay curative treatment.

These observations should be placed in the context of the existing biomarker landscape. Most noninvasive urinary tests for UTUC have been evaluated against benign controls or within UTUC‐only cohorts rather than against RCC.^2^ Reported diagnostic performance varies considerably and direct evaluation in radiologically ambiguous cases that could represent either entity has been largely absent despite being a recurring clinical challenge.^2^, ^6^ A test that performs well precisely in this overlap zone would therefore address a genuine gap. In practical terms, a low BCI in a suspicious lesion could redirect a patient away from URS towards percutaneous biopsy or a renal‐cancer pathway, whereas a high BCI result might support proceeding more confidently to definitive UTUC treatment. Beyond procedural morbidity, avoiding unnecessary interventions could reduce costs and use of scarce hospital resources, including operating‐theatre capacity and inpatient beds.

Several limitations warrant consideration. The cohort was small so confidence intervals around diagnostic estimates are wide and findings should be interpreted cautiously. Nevertheless, despite the limited sample size, BCI values demonstrated a statistically significant separation between UTUC and RCC cases. Further, this was a single‐centre analysis in a predominantly Caucasian population, and the Youden‐derived cut‐off is data‐derived and requires external validation. These results are therefore hypothesis‐generating and should not yet inform clinical decisions independently. Within these constraints, urinary DNA methylation appears to be a promising noninvasive adjunct for differentiating UTUC from RCC when cross‐sectional imaging is inconclusive and warrants evaluation in larger prospective, multicentre studies.

## AUTHOR CONTRIBUTIONS

Christian Daniel Fankhauser had full access to all the data in the study and takes responsibility for the integrity of the data and the accuracy of the data analysis. *Study concept and design*: Ernest Kaufmann, Christian Daniel Fankhauser, Yap Ching Chew and Paolo Piatti. *Acquisition of data*: Ernest Kaufmann, Kilian Röthlin, Christian Daniel Fankhauser, Philipp Baumeister and Agostino Mattei. *Analysis and interpretation of data*: Ernest Kaufmann, Christian Daniel Fankhauser, Yap Ching Chew and Paolo Piatti. *Drafting of the manuscript*: Ernest Kaufmann and Christian Daniel Fankhauser. *Critical revision of the manuscript for important intellectual content*: Ernest Kaufmann, Christian Daniel Fankhauser, Kilian Röthlin, Yap Ching Chew, Paolo Piatti, Philipp Baumeister and Agostino Mattei. *Statistical analysis*: Ernest Kaufmann, Christian Daniel Fankhauser and Yap Ching Chew. *Obtaining funding*: None. *Administrative, technical, or material support*: Ernest Kaufmann, Christian Daniel Fankhauser, Yap Ching Chew and Paolo Piatti. *Supervision*: Ernest Kaufmann and Christian Daniel Fankhauser. *Other*: None.

## CONFLICT OF INTEREST STATEMENT

P. Piatti and Y.C. Chew are employees of Pangea Laboratory and Zymo Research Corp, which provided the Bladder CARE™ test kits. The remaining authors have no conflicts of interest to declare.

## Data Availability

The data that support the findings of this study are available from the corresponding author upon reasonable request.
